# Diagnostic value of chest CT in Iranian patients with suspected COVID-19

**DOI:** 10.22088/cjim.11.0.527

**Published:** 2020

**Authors:** Hanieh Salehi-Pourmehr, Hojjat Pourfathi, Mohammad Kazem Tarzamni, Morteza Ghojazadeh, Behrooz Naghili, Armin Zarrintan, Reza Mahdipour, Sakineh Hajebrahimi

**Affiliations:** 1 Research Center for Evidence-Based- Medicine, Iranian EBM Center: A Joanna Briggs Institute (JBI) Center of Excellence, Tabriz University of Medical Sciences, Tabriz, Iran.; 2 Department of Anesthesiology, Faculty of Medicine, Tabriz University of Medical Sciences, Tabriz, Iran; 3 Department of Radiology, Medical Radiation Sciences Research Group, Imam Reza Hospital, Tabriz University of Medical Sciences, Tabriz, Iran; 4 Research Center for Infectious and Tropical Disease, Tabriz University of Medical Sciences, Tabriz, Iran; 5 Department of Radiology, Imam Reza Hospital, Tabriz University of Medical Sciences, Tabriz, Iran; 6 Department of Urology, Faculty of Medicine, Tabriz University of Medical Sciences, Tabriz, Iran

**Keywords:** Sensitivity, Specificity, Chest CT, RT-PCR, COVID-19

## Abstract

**Background:**

In the current COVID-19 pandemic, there is a rising need for a rapid and reliable diagnostic tool. We hypothesized that chest computed tomography (CT) can be a potential alternative for reverse transcription-polymerase chain reaction (RT-PCR). The aim of this study was to compare the diagnostic value of chest CT and RT-PCR in Iranian patients with suspected COVID-19.

**Methods:**

In a retrospective, single-center case series, 568 consecutive hospitalized or outpatient patients with suspected COVID-19 underwent chest CT and/or RT-PCR testing at Imam Reza Hospital, the tertiary teaching hospital of Tabriz University of Medical Sciences in Iran, from February 21 and March 28, 2020.

**Results:**

The sensitivity of chest CT for signifying COVID-19 was 64% (95% CI: 56%–71%) on the basis of positive RT-PCR results as a standard method. CT imaging also had a specificity of 77% (95% CI: 73%–81%), positive predictive value of 35% (95% CI: 0.31–0.39), negative predictive value of 66% (95% CI: 0.61–0.69), positive likelihood ratio of 2.79 (95% CI: 2.26–3.46), and negative likelihood ratio of 0.47 (95% CI: 0.38–0.57).

**Conclusion:**

Chest CT had higher specificity in the diagnosis of COVID-19 than that of the previous studies. Therefore, it can play a crucial role in the early diagnosis. Similar to the previous studies, the typical CT features were patchy ground-glass opacities as well as peripheral aspects of the lungs consolidations.

Since the World Health Organization has announced the new coronavirus disease (COVID-19) pandemic (1) as a public health emergency (2), early detection and isolation of the infected patients are among the primary importance modalities in the absence of therapeutic methods or specific vaccines to fight the virus. According to the latest clinical guidelines for the diagnosis and treatment of pulmonary inflammation caused by COVID-19, the definitive diagnosis of the disease is made by carrying out reverse transcription-polymerase chain reaction (RT-PCR) test from the bronchoalveolar lavage fluid or blood (3). In the Chinese government guidelines, RT-PCR is the diagnosis standard for COVID-19 pneumonia hospitalization. Since RT-PCR is commonly used because of its easy availability (4), the low sensitivity may lead to misidentification of many infected patients, which can lead to the widespread of this contagious virus. Chest computed tomography (CT), which is routinely used to diagnose pneumonia, could result in fast diagnosis that may be useful for diagnosing COVID-19. One of the specific pattern of COVID-19 in radiography is multifocal, bilateral and peripheral, or in the early phase of disease, unifocal ground-glass opacities. The other features include multifocal patchy consolidations and/or interstitial changes. The mentioned features may be present in symptomatic cases with negative RT-PCR results (3, 4). 

The current study aimed to compare the diagnostic accuracy of chest CT and RT-PCR in Iranian patients with suspected COVID-19. This retrospective, single-center case series study of 568 consecutive hospitalized or outpatient suspected COVID-19 cases was approved by the local Ethics Committee of Tabriz University of Medical Sciences, Tabriz, Iran (Code: IR.TBZMED.REC.1398.1276). 

Considering retrospective design of the study, consent process was not undertaken. Patients with the sign or symptoms of COVID-19 including cough, fever, and dyspnea who had chest CT and/or RT-PCR assay using throat swab samples in the tertiary teaching hospital of Imam Reza of Tabriz University of Medical Sciences in Iran were enrolled respectively. Both chest CT scan and RT-PCR were taken on the day of admission of the patients with suspected COVID-19. The RT-PCR results of patients were gathered from electronic medical records of information system of the Imam Reza Hospital. After specimen collection, the throat swabs were put into the special tubes containing 150 μL of virus preservation solution. In 2 hours total RNA was extracted by means of a respiratory sample RNA isolation kit (RT-PCR test kit; Sansure biotech). For chest CT imaging, patients were in a supine position using a SOMATOM Emotion 6 scanner (Siemens Healthineers, Germany). The scanning parameters were: tube voltage, 110 kVp; automatic tube current modulation, 30–70 mAs; pitch, 1.45 mm; matrix, 512 × 512; slice thickness, 8 mm; and field of view, 350 mm × 350 mm. Finally, every image having the same increment was reconstructed by a slice thickness of 0.625 mm to 1.250 mm. A radiologist interpreted the chest CT images while he was blinded to patients’ RT-PCR results and categorized them as negative or positive for COVID-19. The main chest CT pattern was multifocal, bilateral and peripheral, or in the early phase of disease, unifocal ground-glass opacities. The other features included multifocal patchy consolidations and/or interstitial changes in the left lung, right, lung, or bilateral. 

**Statistical analysis: **RT-PCR results was used as reference standard test to determine the sensitivity, specificity, positive predictive value (PPV), and negative predictive value (NPV) of chest CT images. 

## Results

The data of 568 suspected COVID-19 cases from February 21 to March 28, 2020, of 568 patients with suspected COVID-19, showed that 314 (55.3%) were males. There was no statistically significant difference in the number of cases between genders (P=0.343). The median age of cases was 58 years (interquartile range: 41–71 years). The results showed that RT-PCR test results of 201(35.4%) patients were positive, and 174 (30.6%) chest CT scans were also positive (abnormal CT findings consistent with viral pneumonia). 

Using the chi-square test, the results showed that the majority of patients (82.8%, 304 of 367) with negative RT-PCR results had negative CT scans. On the other hand, 17.2% of patients (63 of 367) had positive chest CT findings. Furthermore, 55.2% of cases (111 of 201) who had positive RT-PCR results had positive chest CT findings as well, and also the other 90 patients had no CT features suggestive of COVID-19. The consistency of the two tests' results was statistically significant (P<0.001). Based on the results of RT-PCR (as a standard method), chest CT had 64% sensitivity in diagnosis of COVID-19. Besides, the chest CT had a specificity of 77.2% (table 1). Our results showed a NPV of 66% (95% CI: 0.61–0.69) and positive likelihood ratio (LR+) of 2.79 (95% CI: 2.26–3.46). 

**Table 1:** Sensitivity, specificity, PPV, NPV, LR+, LR-, and for chest CT scans in comparison to RT-PCR for COVID-19

**CT Scan**	**Sensitivity (95% CI)**	**Specificity** **(95% CI)**	**PPV** **(95% CI)**	**NPV** **(95% CI)**	**LR+** **(95% CI)**	**LR-** **(95% CI)**
	64% (0.56-0.71)	77% (0.73-0.81)	35% (0.31-0.39)	66% (0.61-0.69)	2.79 (2.26-3.46)	0.47 (0.38-0.57)

**Figure 1.**
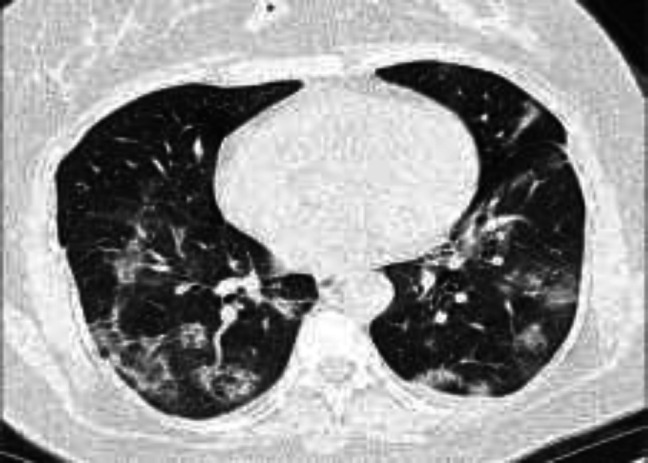
A 50-year-old male who presented with fever and cough showed bilateral multifocal patchy ground-glass opacities, which were more prominent in the lower, peripheral, and posterior zones

**Figure 2:**
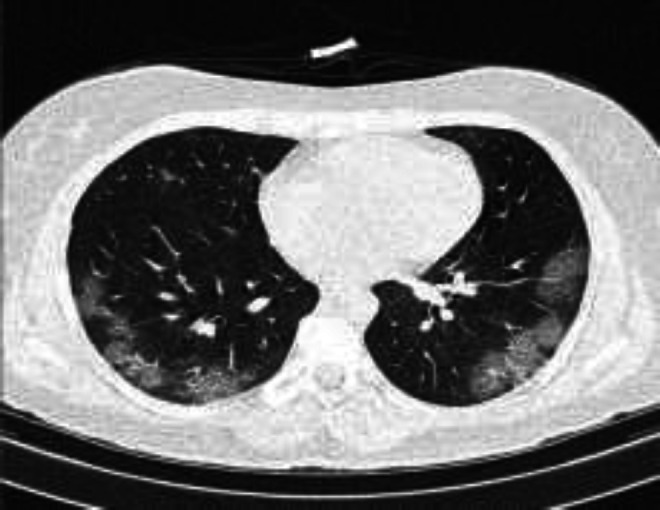
A 56-year-old female with complaint of fever and dyspnea and ground-glass opacities in both lungs

**Figure 3:**
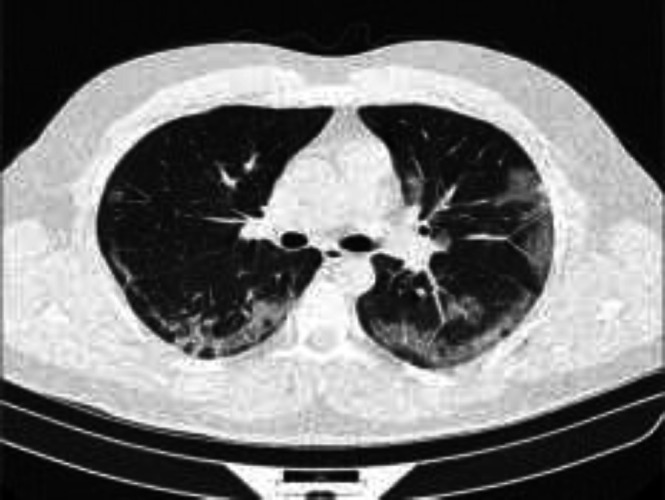
A 62-year-old female patients with chief complaint of dyspnea, fever, and cough showing bilateral ground-glass opacities in chest CT

Similar to the results of previous studies, the typical CT features were patchy ground-glass opacities and large consolidations in the peripheral parts of the lungs (figures 1, 2, 3). The results of death in the case of confirmed COVID-19 by RT-PCR or CT-scan were compared and revealed that 35.3 % of patients with positive results of PCR died. While this rate was 27.9% for patients with a positive chest CT scan (table 2).

**Table 2:** The rate of death in confirmed COVID-19 cases

**Crosstab**				
Death Status	**CT Result**		**PCR Results**	
	**Negative**	**Positive**	**Negative**	**Positive**
Count % within Status	49	19	44	24
	72.1%	27.9%	64.7%	35.3%

## Discussion

While the origin of COVID-19 is still being investigated, the diagnosis and isolation of infected patients at the early stage of the disease is an essential step in the prevention of the disease spread (5). Currently, RT-PCR of sputum samples, throat swabs, and lower respiratory tract secretions are used to diagnose COVID-19 (6). However, RT-PCR has a low sensitivity (ranging between 45% and 60%), which may be due to low viral load, incorrect sampling methods, inaccurate sample source, inappropriate sampling time (upper respiratory tract samples have peak viral loads 3 days after the start of symptoms), nucleic acid detection technology insufficiencies, variations in the detection rates of manufactured kits (considering that performing the test requires at least several hours), and notable false negative rates. Chest CT on the other hand, can help rapidly screen patients infected with COVID-19 (7). 

In our study, the specificity of chest CT (77%) was greater than that of RT-PCR. Similar to previous reports, the most common chest CT features were ground-glass opacities. Previous studies demonstrated a greater sensitivity of chest CT in diagnosis of COVID-19 compared to RT-PCR in early phase (3, 8). Fang et al. reported a positive PCR result rate of 70% after a single respiratory swab, 94% cumulatively after a second swab test, and 98% cumulatively after a third swab test. They reported abnormal chest CT findings too which were consistent with viral pneumonia in 98% of patients. Hence, they suggested that CT images were more sensitive than PCR (8). 

Concordant results (positive PCR results and positive CT scan findings) were demonstrated only in 55.2% of patients in this study. This result conflicts with a previous study in which concordant results were demonstrated in 93% of patients. Furthermore, in the current study, discordant results (positive PCR results but negative CT scan findings) were observed in 44.8% of patients. However, a previous study reported discordant results in 4% of patients (9). This difference may be the result of the small sample size of the previous study.

In this study, the RT-PCR positive result rate in detection of COVID-19 was 35.4%, which was consistent with previous reports (30%–60%) (10). In the study by Ai et al., the RT-PCR positive result rate was 59%. This rate was 88% for chest CT and chest CT had 97% sensitivity (3). However, the current study showed lower sensitivity of 64% for chest CT. Both PCR and chest CT scan were obtained on the day of admission of the patienst with suspicious COVID-19 signs and symptoms. The chest CT may be negative false within the beginning phase of the disease, and as we have studied the chest CT of the primary day of patients, it might account for the low sensitivity of chest CT exam. Also, our investigations showed higher specificity for chest CT. Our results support the use of chest CT as a rapid, reliable, validated, and widely available method for screening patients with clinical features of infection with COVID-19. Our results are in accordance with a previous study that suggested using diagnostic algorithms based on a combination of RT-PCR results and chest CT scan findings to ensure accurate detection of disease in hospitalized patients (9). 

In this study, 55.2% of the patients with COVID-19 confirmed by RT-PCR tests presented a positive chest CT finding as well that was lower than the results described in previous studies (3, 7). The retrospective design of our study gave rise to certain limitations including unequal time between CT scans obtained for each patient, no evaluations for pathological changes, and data missing from the patient’s hospital records.

Our results showed higher specificity with CT imaging in diagnosing COVID-19 than that of the previous studies. CT imaging may play a crucial role in the diagnosis of COVID-19 at the early phase which is essential for appropriate control and treatment of the disease. Patchy ground-glass opacities and peripheral parts of the lungs consolidations are the typical features of COVID-19 patient’s CT images. 

## References

[1] World Health Organization Infection prevention and control during health care when novel coronavirus (nCoV) infection is suspected. https://www.who.int/publications/i/item/infection-prevention-and-control-during-health-care-when-novel-coronavirus-(ncov)-infection-is-suspected-20200125.

[2] Wang D, Hu B, Hu C (2020). Clinical characteristics of 138 hospitalized patients with 2019 novel coronavirus-infected pneumonia in Wuhan, China. JAMA.

[3] Ai T, Yang Z, Hou H (2020). Correlation of Chest CT and RT-PCR Testing in Coronavirus Disease 2019 (COVID-19) in China: A Report of 1014 Cases. Radiol.

[4] El-Tholoth M, Bau HH, Song J ( 2020). A single and two-stage, closed-tube, molecular test for the 2019 Novel Coronavirus (COVID-19) at home, clinic, and points of entry. ChemRxiv.

[5] Guo YR, Cao QD, Hong ZS (2020). The origin, transmission and clinical therapies on coronavirus disease 2019 (COVID-19) outbreak–an update on the status. Mil Med Res.

[6] Xia J, Tong J, Liu M, Shen Y, Guo D (2020). Evaluation of coronavirus in tears and conjunctival secretions of patients with SARS-CoV-2 infection. J Med Virol.

[7] Xie X, Zhong Z, Wei Z (2020). Chest CT for typical 2019-nCoV pneumonia: relationship to negative RT-PCR testing. Radiology.

[8] Fang Y, Zhang H, Xie J (2020). Sensitivity of chest CT for COVID-19: comparison to RT-PCR. Radiology.

[9] Al-Tawfiq JA, Memish ZA (2020). Diagnosis of SARS-CoV-2 infection based on CT scan vs RT-PCR: reflecting on experience from MERS-CoV. J Hospital infection.

[10] Yang Y, Yang M, Shen C ( 2020). Evaluating the accuracy of different respiratory specimens in the laboratory diagnosis and monitoring the viral shedding of 2019-nCoV infections. medRxiv.

